# Tannin-Rich Plants as Natural Manipulators of Rumen Fermentation in the Livestock Industry

**DOI:** 10.3390/molecules25122943

**Published:** 2020-06-26

**Authors:** G. M. Fagundes, G. Benetel, K. C. Santos, K. C. Welter, F. A. Melo, J. P. Muir, I. C. S. Bueno

**Affiliations:** 1 Department of Animal Science, Universidade Federal de Roraima–UFRR, BR 174, km 12, 69300-000 Boa Vista, Roraima, Brazil; 2 Department of Animal Science, Universidade de São Paulo–USP, Av. Duque de Caxias Norte, 225, 13635-900 Pirassununga, São Paulo, Brazil; gabriela.benetel@usp.br (G.B.); katielicarolinewelter@usp.br (K.C.W.); flavia.melo@usp.br (F.A.M.); ivesbueno@usp.br (I.C.S.B.); 3 Department of Animal Science, Universidade Federal Rural de Pernambuco–UFRPE, Av. Dom Manuel de Medeiros, 52171-900 Recife, Pernambuco, Brazil; kelly_venturosa@hotmail.com; 4 Texas A&M AgriLife Research, 1229 North U.S. Hwy 281, Stephenville, TX 76401, USA; Jim.Muir@ag.tamu.edu

**Keywords:** bioactive compounds, cattle, legumes, sustainable

## Abstract

Condensed tannins (CTs) are plant anti-herbivore compounds with antimicrobial activity that can be used in ruminant diets as ruminal microbiome manipulators. However, not all CTs from fodder legumes are bioactive due to their wide structural diversity. The aim of our study was to investigate the effect of 10 CT-containing plants *(Flemingia macrophylla*, *Leucaena leucocephala*, *Stylosanthes guianensis*, *Gliricidia sepium*, *Cratylia argentea*, *Cajanus cajan*, *Desmodium ovalifolium*, *Macrotiloma axilare, D. paniculatum*, and *Lespedeza procumbens*) on in vitro fermentation kinetics of Nelore beef cattle. Polyethylene glycol (PEG), a specific CT-binding agent, was added to neutralize condensed tannin. Tifton and alfalfa hay were used as controls lacking CT. The experimental layout included a randomized complete block with factorial design and four blocks. The data were subjected to analysis of variance followed by Duncan’s test to determine differences (p < 0.05) among treatment means. The addition of PEG in browse incubations resulted in increased gas production, fermentation rate, short-chain fatty acid (SCFA) and N-NH_3_ release. Within our study, *Lespedeza procumbens*, *Desmodium paniculatum*, *Leucaena leucocephala*, *Desmodium ovalifolium*, and *Flemingia macrophylla* showed superior bioactivity compared to other species evaluated, suggesting a natural alternative for replacing ionophores to modify ruminal fermentation. Condensed tannins from *L. pocumbens*, *D. paniculatum*, *L. leucocephala*, *D. ovalifolium*, and *F. macrophylla* have the potential to modify rumen fermentation in beef cattle.

## 1. Introduction

In tropical and subtropical environments, such as Brazil, grasses of African origin are the most pervasive pasture species for cattle grazing [1]. Because of poor digestibility and high C:N ratios, this led to low livestock productivity and increased rumen methane emissions in these climates [2,3]. In addition, Nelore, a Zebu breed that has less feed efficiency and emits more methane [2], is the predominant beef cattle in Brazilian livestock. This combination led to the search for legumes and their byproducts that can be added to diets of cattle grazing cultivated grass pastures to mitigate greenhouse gas emissions without compromising livestock productivity [4,5].

Many tropical and temperate plant species have chemical defenses, such as condensed tannins (CTs), which can complex with proteins and other macromolecules, providing bactericidal effects [3,6]. Warm-climate arboreal, brush, and herbaceous legumes were long proposed as tropical and subtropical pasture forages that provide protein to livestock and their rumen microbiome; many are also rich in CTs [7]. Legumes containing CTs possess antimicrobial properties capable of manipulating rumen microbiomes. This biodiversity could become a significant alternative for improving sustainable productivity in ruminants grazing tropical and subtropical pastures [3,8]. In primarily tropical and subtropical countries with an expanding agricultural sector, such as Brazil, the use of legume forages is a vital tool for sustainable management in livestock production because they increase dietary protein, reduce greenhouse gas emissions, and improve livestock health in myriad ways.

However, the biological properties of tropical and subtropical forage legume phenolic compounds depend on the level and type in the plant [5,9]. Knowledge of not only phenolic composition but also their effect on ruminal fermentation kinetics is needed for each individual species if we are to fully harness their potential to improve livestock production [3]. The objective of our study was to investigate the effects of 10 CT-rich legumes on in vitro fermentation kinetics in Nelore beef cattle.

## 2. Results

In vitro degradability differed among plants and PEG treatments (Table 1). Both control Tifton and alfalfa showed greater IVDMD and IVOMD compared to species containing CT. Among tannin-rich legumes, *S. guianensis*, *G. sepium*, and *M. axilare* had higher degradability, followed by *L. leucocephala* and *C. argentea* with intermediate values. *Lespedeza procumbens*, followed by *D. paniculatum* and *F. macrophylla*, had low in vitro degradability compared to other entries; however, when PEG was added, the degradability of these plants increased.

The partitioning factors in *C. argentea*, *D. paniculatum*, *F. macrophylla*, and *L. procumbens* obtained without PEG were greater than those obtained after adding PEG. The addition of PEG had no significant effect on the partitioning factor in other species.

Tannins influenced the concentrations of N-NH_3_ in the rumen, which were lower without PEG. *Desmodium ovalifolium*, *D. paniculatum*, *F. macrophylla*, *L. procumbens*, and *L. leucocephala* had lower concentrations of N-NH_3_ with tannin incubations (non-treated PEG) compared to other entries.

Only *D. paniculatum* and *L. procumbens* had lower levels of acetate and propionate when PEG was not used. Total tannins in most plants reduced butyrate concentrations (Table 2). Tannin-containing plant such as *D. ovalifolium*, *D. paniculatum*, *F. macrophylla*, and *L. procumbens* had higher levels of butyrate with PEG incubations. However, the effect of tannin was more pronounced on isobutyric, isovaleric, and valeric acid levels where, with the exception of the controls, *G. sepium*, *M. axilare*, and *S. guianensis* had lower levels of isoacids than those with PEG addition.

When assessing the parameters of France et al. (1993) [10] (Table 3), the maximum increase in potential of gas production (*A*) after 96 h of fermentation was recorded in Tifton grass (control) with and without PEG addition, followed by *S. guianensis*, alfalfa, and *M. axilare*. Reactive TC from *D. paniculatum*, *L. procumbens*, and *F. macrophylla* resulted in low values of *A* in incubations without PEG. When tannin was bound with PEG, with the exception of *G. sepium*, the levels of *A* increased in all tannin-containing plants. However, only *L. procumbens* had increased lag time (*L*) as a result of an interaction between the plant species and PEG. In *D. ovalifolium*, *F. macrophylla*, and *L. procumbens*, the time required to reach half of the asymptotic value (*T/2*) was greater than when PEG neutralized CT.

**Figure 1:**
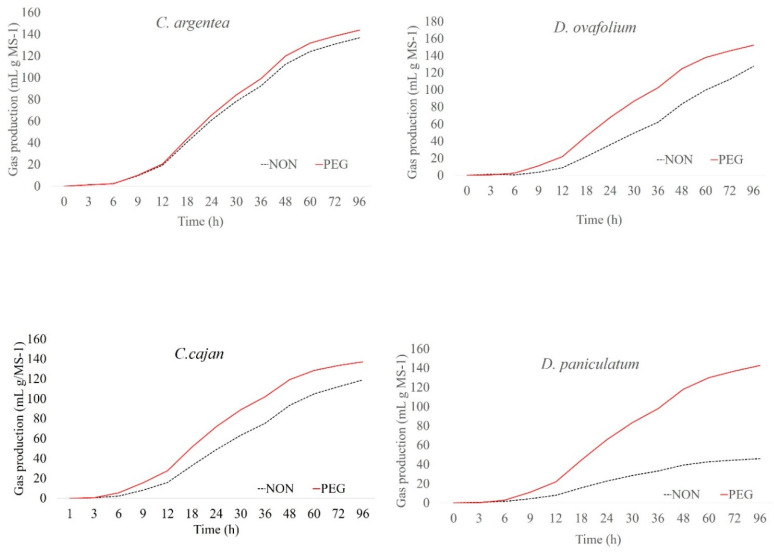
Cumulative gas production curves of *Cratylia argentea*, *Cajanus cajan*, *Desmodium ovalifolium*, and *Desmodium paniculatum* incubated at the end of 96 h.

**Figure 2:**
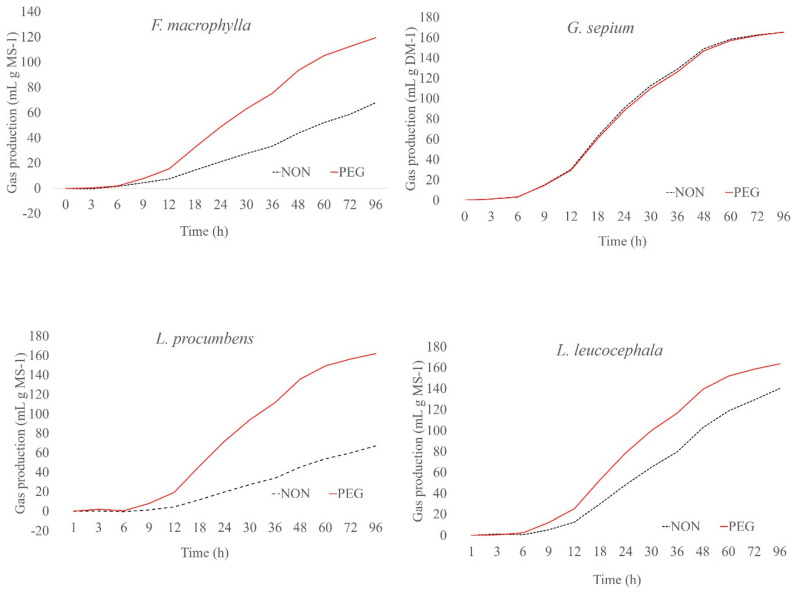
Cumulative gas production curves of *Flemingia macrophylla*, *Gliricidia sepium*, *Lespedeza procumbens*, and *Leucaena leucocephala* incubated at the end of 96 h.

**Figure 3:**
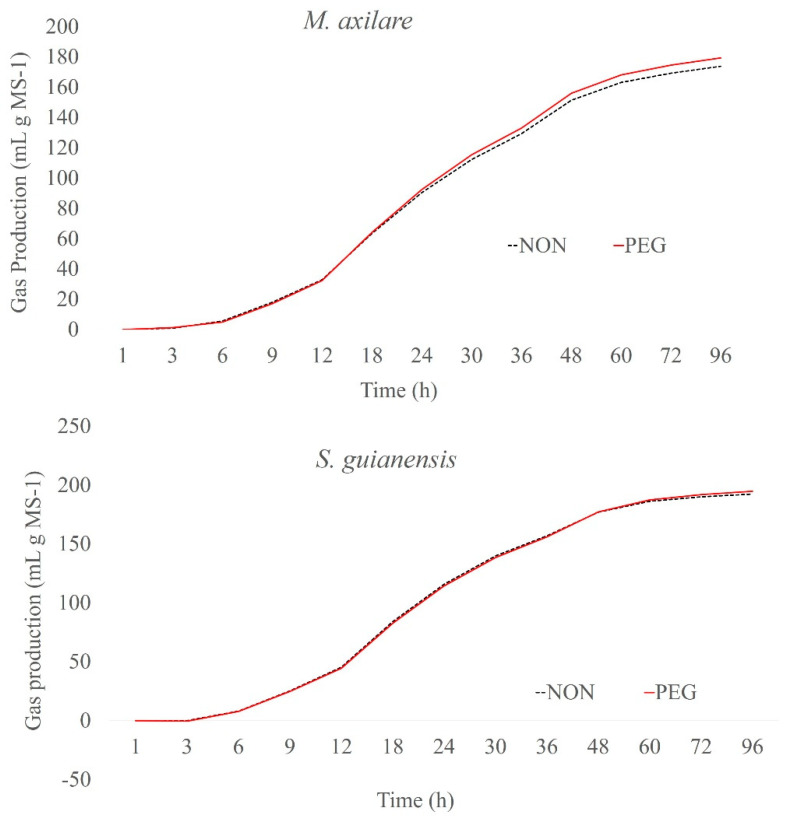
Cumulative gas production curves of *Macrotiloma axilare* and *Stylosanthes guianensis* incubated at the end of 96 h.

**Figure 4:**
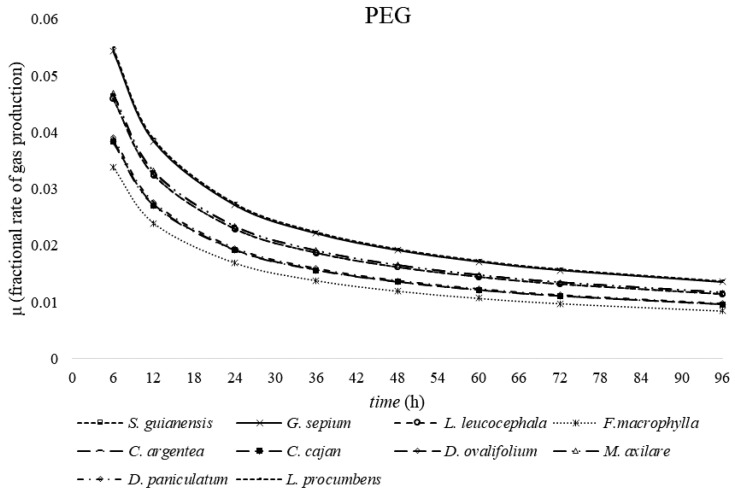

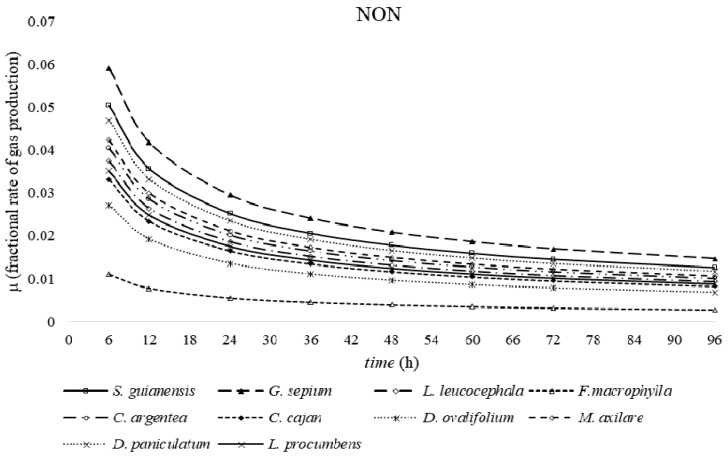
Gas production curves of legumes forages incubated at the end of 96 h.

The cumulative gas production curves from plant samples after 96 h of fermentation are shown in Figure 1, Figure 2 and Figure 3. Among the plants, *G. sepium* and *S. guianensis* had similar fermentation with and without PEG. For *C. argentea* and *M. axilare*, the fermentation process was slightly increased with PEG addition. An increase in gas production obtained after incubation with PEG treatment was recorded for *C. cajan*, *D. ovalifolium*, and *L. leucocephala*. However, the maximum effect of tannins on gas suppression was found in *D. paniculatum*, *F. macrophylla*, and *L. procumbens*, whereas, in the presence of PEG, these values were greater. The inclusion of PEG for *L. procumbens*, *F. macrophylla*, *D. ovalifolium*, *D. paniculatum*, and *L. leucocephala* resulted in improved gas production rates (μ) (Figure 4). However, adding PEG did not improve μ values in *G. sepium* and *S. guianensis.*

## 3. Discussion

The main goal of our study was to identify, using an in vitro gas technique, tannin-rich plants as a natural alternative to manipulate rumen microbial fermentation for methane reduction in Nelore beef cattle. Thus, by assessing data from browse fermentation kinetics, we clearly observed that the CT content in *L. procumbens*, *D. paniculatum*, *L. leucocephala*, *D. ovalifolium*, and *F. macrophylla* had high reactivity among the tested legumes. Their different kinetic behavior in the presence of PEG confirmed the influence of CT on the fermentation process. However, fermentation characteristics in *G. sepium*, *S. guianensis*, and *M. axilare* were similar with or without PEG added during incubation, reflecting no biological effect of CT in these samples. The greatest effect on the cumulative gas production was recorded in *D. paniculatum*, although it did not have the highest CT content. The fact that some CTs are more reactive than others is related to their chemical structure as much as to their CT concentration [9,11]; thus, our work highlights the importance of studies that predict CT reactivity in ruminant nutrition rather than simply its quantity.

Rumen degradability and kinetic characteristics were affected by browse samples and PEG treatment. As expected, the least reactive plant species (*G. sepium*, *S. guianensis*, and *M. axilare*) showed maximum IVDMD and IVOMD, and the most reactive browse samples (*D. paniculatum*, *L. procumbens*, and *F. macrophylla*) had minimal IVDMD and IVOMD. An improvement in IVDMD and DIVMO after PEG incubation was observed in our study, reflecting the inhibitory effect of CT on rumen microbial degradability. This finding is consistent with previous studies reported in the literature [12,13].

We observed that the most reactive plant samples, when incubated without PEG, resulted in high PF values (DMD/GP), whereas, in the presence of PEG, the same did not occur. This suggests greater gas production and lower herbage fiber degradation after CT neutralization. Similar results were reported by Bueno et al. (2015) [2], who also recorded high levels of PF during incubation of herbage containing CT; those authors reported that, unlike true degradability, if apparent degradability was measured, the losses of washable fractions may result in inaccurate FP values. Therefore, we hypothesize that the high PF values in our assay are a consequence of soluble fraction losses from substrates due to the washing process; the degradation of these substrates was inhibited by CT in the rumen medium. Such effects, therefore, lead us to affirm that PF is not an adequate parameter to measure microbial efficiency in CT-containing herbage trials, leading to incorrect estimates.

Because of CT–protein complexation, incubation without PEG resulted in lower concentrations of ruminal ammonia. The relatively low values of N-NH_3_ in *D. ovalifolium*, *D. paniculatum*, *L. procumbens*, *F. macrophylla*, and *L. leucocephala* incubations are probably due to inhibition of amino-acid deamination by ruminal microorganisms. The decrease in N-NH_3_ concentrations obtained in incubations with tannin-containing plants is a phenomenon of CT previously reported in in vitro assays [14,15]. This was also observed in our in vivo studies (Fagundes et al., unpublished data), in which goats fed *Flemingia* had decreased ammonia values compared to those fed a diet with PEG inclusion.

Although the most pronounced reduction in the presence of tannins constituted isoacid concentrations, in the absence of PEG, fermentation of *D. ovalifolium*, *D. paniculatum*, *F. macrophylla*, and *L. procumbens* resulted in a decrease in butyrate levels. In two samples, *D. paniculatum* and *L. procumbens*, a decrease in acetate concentration was also observed. Condensed tannins can alter ruminal microbiota profile and, depending on their structure, molecular weight, and type of binding to macromolecules, they can selectively affect specific rumen bacteria and consequently alter the metabolic processes of short-chain fatty acids in the rumen [16]. Several authors [15,17] found similar results.

As expected, compared with legume samples, the control Tifton, due to its higher fiber content, had the greatest potential gas production (*A*). Similar to the alfalfa (*Medicago sativa*) control, the least reactive species (*S. guianensis*, *M. axilare*, and *G. sepium*) had greater values of *A*. When samples were incubated with PEG, the increase in potential gas production (*A*) was greater in almost all plants, indicating inhibition of substrate degradation by CT. The relative decrease in potential gas production (*A*) in *D. paniculatum*, *L. procumbens*, and *F. macrophylla* in the absence of PEG reflects their superior reactivity compared to other plants. Likewise, in relation to the France (1993) [10] model, tannins from *D. procumbens*, *L. procumbens*, and *F. macrophylla* had decreased asymptotic time (*T/2*). Furthermore, CT resulted in a greater time of colonization (*L*) only in *L. procumbens*; this was probably because highly bioactive CT affected the microbial fermentation of soluble and readily available fractions in the rumen. This agrees with the reports of Baba et al. (2002) [12], which concluded that the increase in gas production not accompanied by a proportional increase in substrate degradability in PEG incubations reflects an improvement in degradation of both soluble and insoluble fractions.

Tannins also influenced fermentation rate and gas production after 96 h of incubation. In the absence of PEG, the greatest reductions in the gas production curve were recorded in *D. paniculatum*, *F. macrophylla*, and *L. procumbens*. Compared with non-PEG treatment, PEG addition during incubation of *D. ovalifolium* and *F. macrophylla* resulted in greater fermentation rates (μ). Studies by Kamalac et al. (2005) [18] and Calabrò et al. (2012) [19] also reported an increase in GP content with the addition of PEG.

## 4. Materials and Methods

### 4.1. Tannin-Rich Plants

Herbage material was collected from tropical (Flemingia macrophylla, Leucaena leucocephala, Stylosanthes guianensis, Gliricidia sepium, Cratylia argentea, Cajanus cajan, Desmodium ovalifolium, and Macrotiloma axilare) and temperate (Desmodium paniculatum and Lespedeza procumbens) CT-rich species. Legumes from tropical environments were obtained at the Embrapa Agrobiologia and Universidade Federal Rural of Rio de Janeiro in southeastern Brazil, in the municipality of Seropédica, Rio de Janeiro Brazil (22°44′38″ south (S); 43°42′28″ west (W); altitude 33 m). The temperate plants were obtained at the Texas AgriLife Research Center, Stephenville TX, United States of America (USA) (32°13′13″ north (N); 98°12′49″ W; 388 m altitude). Hay of Tifton 85 grass (Cynodon sp.) and alfalfa (Medicago sativa) harvested at Stephenville TX USA were used as controls because they contain little CT.

The herbaceous legumes were harvested close to the ground and assayed as whole plants. Leaves and tender shoots from trees and shrubs were hand-clipped to a height of 1.00 m, from 0.5 × 0.5 m quadrants at five different points. The samples obtained were mixed by plot, subsampled, dried at 40 °C (to avoid inactivating CT) in forced ventilation, and ground to pass a 1 mm sieve for all assays except phenols which were assayed on samples that passed through a 0.25 mm sieve.

### 4.2. Chemical Analysis

All dried samples were analyzed for dry matter (DM) (ID 930.15), mineral matter (MM) (ID 942.05) by combustion at 450 °C, crude protein (CP) (ID 954.01) by micro-Kjeldhal method using a conversion factor of 6.25, and ether extract (EE) (ID 920.39) by exhaustive extraction with ether [20], while neutral-detergent fiber (NDF-NDF; ID 973.18) and acid-detergent fiber (ADF) were determined according to Mertens (2002) [21], sequentially with the addition of α-amylase and inclusion of residual ash. Acid detergent lignin (ADL) was determined by solubilization of cellulose with sulfuric acid as described by Robertson and Van Soest (1981) [22]. Total phenol (TP) concentrations were quantified by the Folin–Ciocalteu reagent method [23], while total tannins (TTs) were estimated as the difference in TP concentration before and after treatment with insoluble polyvinylpolypirrolidone [24], using tannic acid as the standard. Concentrations of extractable, protein-bound, fiber-bound, and total CT were determined using the HCl–butanol method based on photospectrometric measurements as described by Terrill et al. (1992) [25]. Species-specific standards were created for each plant species analyzed [26] using CT extracts purified on Sephadex LH-20 and lyophilized to recover purified CT. All compositional data were determined in duplicate and are reported on a DM basis. The chemical compositions of the plants are presented in Table 4.

### 4.3. Animals and Inoculum Preparation

All methods and animal care were performed in accordance with the relevant guidelines and regulations of the Ethic Committee on Animal Use of the School of Animal Science and Food Engineering, (São Paulo University) (protocol number CEUA 2416120916).

Ruminal contents from four adult rumen-cannulated Nellore cattle grazing a tropical grass pasture were collected individually before the morning feeding. The solid phase was collected manually and stored in heated boxes at 39 °C, while the liquid phase was collected by using a vacuum pump and stored in pre-warmed thermal bottles, previously flushed with CO_2_. One inoculum was prepared for each donor animal (blocks). For each inoculum, the phases were homogenized in a 1:1 ratio in a blender for 10 s and filtered through three layers of cotton cloth, according to Bueno et al. (2005) [27]. The inocula were constantly saturated with CO_2_ and held in a water bath (39 °C) until use.

### 4.4. In Vitro Gas Production Assay

The effect of tannin-rich plants on fermentation kinetics was obtained using a pressure transducer according to the semi-automatic in vitro gas production technique proposed by Theodorou et al. (1994) [28] and modified by Mauricio et al. (1999) [29]. Two sub-samples of each plant (0.5 g) were placed into 160mL glass bottles in duplicate, combined or not with a CT-binding agent (polyethylene glycol; PEG 6000, Synth, Diadema, Brazil) to neutralize CT. Then, 0.5 g of PEG was added to the sample directly into the fermentation flasks. Four flasks were prepared for each sample: two with no PEG and two with PEG. The inoculation was performed by injecting 25 mL of inoculum in 50 mL of buffered mineral solution (Menke’s buffered medium) [30] into each fermentation flask. Blanks were used for each inoculum to measure the fraction of total gas production due to substrate in inocula, and these values were subtracted from the total to obtain net GP. The flasks were sealed with butyl rubber septum stoppers, manually shaken, and kept in a forced-ventilation oven at 39 °C for 96 h. All legume samples were incubated simultaneously in the run. After 3, 6, 9, 12, 18, 24, 30, 34, 48, 60, 72, and 96 h, the pressure inside each bottle was measured using a transducer (Pressure Press Data 800; Piracicaba, SP, Brazil), and those values were used to estimate the gas volume produced. The values obtained were transformed into volume by the following equation defined for the test laboratory conditions: *V* = *p* × 4.6788; where *V* = gas volume (mL) and *p* = pressure (psi). At the end of the incubation (96 h), the bottle was opened and samples of the ruminal liquid contained in each glass bottle were collected for determination of short-chain fatty acids and ammoniacal *N* (*N*-NH_3_). The remaining material was filtered in crucibles to determine in vitro apparent DM degradability (IVDMD) and in vitro apparent organic matter degradability (IVOMD). The partition factor (PF) was determined by the ratio between the dry matter degradation (DMD; mg) and gas production (GP; mL), according to Blümmel et al. (1997) [31].

Gas production data were used to determine the fermentation kinetics based on the model of France et al. (1993) [10].

(1) $$y=A\;\{1-e^{\left[-b\left(t-L\right)-c\left(\surd t-\surd L\right)\right]}\},$$

(2) μ=-(b+c)/(2√t),

where y = cumulate gas production (mL/g DM) in incubation time t (h), A = potential gas production (mL/g DM), *L* = lag time (h), b and c are constants of the model, *T/2* = time to half-asymptote (h), and μ = fractional rate of gas production (h^−1^). The kinetic parameters (*A*, *L*, μ, and *T/2*) were compared in the statistical analysis.

### 4.5. Short-Chain Fatty Acid Determination

The short-chain fatty acid profiles in ruminal liquid were determined by gas chromatography (GC-2014, Shimadzu^®^, Tokyo, Japan) as described by Bueno et al. (2020) [32]. Ruminal samples were centrifuged at 14,500× *g* for 10 min, and the supernatant (800 µL) was transferred to a flask with 200 µL of formic acid (98–100%) and 100 µL of the internal standard (100 mM 2-ethyl butyric acid, Chem service, USA). Acetic, propionic, isobutyric, butyric, isovaleric, and valeric acids (99.5% purity, Chem service, USA) were used as quantitative external standards. The following chromatographic conditions were employed throughout this study: injector and detector temperatures, 250 °C; hydrogen flow to the flame jet at 60 kPa; helium carrier gas at 8.01 mL/min; synthetic air at 40 kPa.

### 4.6. N-NH_3_ Determination

N-NH_3_ concentrations in the ruminal liquid were obtained by distillation (2 mL sample) with 50% potassium hydroxide (KOH) and titration with 0.05 N sulfuric acid (micro-Kjeldahl method), according to the methodology described by Preston (1995) [33].

### 4.7. Experimental Design and Statistical Analysis

The experimental design included a randomized complete block with factorial design (12 forages with and without PEG) and four repetitions (blocks/inoculum donors), according to the following model:

Y_ijk_ = μ + α_i_ +β_j_ + (ab)_ij_ + b_k_ + e_ijk_, (3)

where Y_ijk_ is the dependent variable, μ is the overall mean, α_i_ = effect of legumes (substrates) (i = 1 to 12), β_j_ = effect of PEG addition or not (j = 1 to 2), (ab)ij = interaction of substrates and PEG addition, b_k_ = effect of ruminal inoculum (k = 1 to 4), and e_ijk_ = random variation.

Gas production differences were compared using the non-linear procedure of SAS 9.2 Program (Institute Inc., Cary, NC, USA) [34]. Data were subjected to analysis of variance followed by a Duncan’s test to determine the significance of the difference between treatment means. Unless otherwise noted, probabilities were considered significant at p ≤ 0.05.

## 5. Conclusions

The neutralization of CT by adding PEG to incubations of herbage containing biologically active CT resulted in increased gas production, fermentation rate, AGCC, and N-NH_3_, showing that CT clearly affected the in vitro fermentative kinetics of these species in the rumen. Condensed tannins from *L. procumbens*, *D. paniculatum*, *L. leucocephala*, *D. ovalifolium*, and *F. macrophylla* were effective in modifying ruminal fermentation, which indicates a promising alternative to ionophores for methane reduction in beef cattle. We suggest, therefore, further research to map the main monomer components of these plant secondary compounds and to document their effects as ruminal manipulators.

## Figures and Tables

**Table 1:** Effects of tannins from plant species on ruminal degradability, microbial efficiency and *N*-NH_3_ at 96 h of incubation in vitro.

Variables/Plant			
**IVDMD (g/kg DM)**	**Without PEG**	**With PEG**	**Plant × PEG ^(2)^**
*Cajanus cajan*	403 ^§D^	435 ^DE^	ns
*Cynodon* spp (Tifton 85)	751 ^A^	739 ^A^	ns
*Cratylia argentea*	553 ^aC^	458 ^bD^	***
*Desmodium ovalifolium*	432 ^D^	474 ^D^	ns
*Desmodium paniculatum*	278 ^bF^	336 ^aF^	*
*Flemingia macrophylla*	300 ^bF^	362 ^aF^	*
*Gliricidia sepium*	676 ^B^	633 ^C^	ns
*Lespedeza procumbens*	352 ^bE^	406 ^aE^	*
*Leucaena leucocephala*	567 ^bC^	633 ^aC^	*
*Medicago sativa*	697 ^B^	703 ^BC^	ns
*Macrotiloma axilare*	670 ^B^	671 ^BC^	ns
*Stylosanthes guianensis*	694 ^B^	695 ^AB^	ns
*SEM* ^(1)^	2.42	2.10	1.81
p-values ^(2)^	***	***	
**IVOMD (g/kg DM)**	**Without PEG**	**With PEG**	**Plant × PEG**
*Cajanus cajan*	406 ^§D^	438 ^CDE^	ns
*Cynodon* spp (Tifton 85)	753 ^A^	748 ^A^	ns
*Cratylia argentea*	554 ^C^	502 ^C^	ns
*Desmodium ovalifolium*	429 ^D^	471^CD^	ns
*Desmodium paniculatum*	273 ^bF^	409 ^aDE^	***
*Flemingia macrophylla*	298 ^F^	357 ^E^	ns
*Gliricidia sepium*	685 ^B^	637 ^B^	ns
*Lespedeza procumbens*	357 ^E^	409 ^DE^	ns
*Leucaena leucocephala*	557 ^bC^	628 ^aB^	*
*Medicago sativa*	689 ^B^	697 ^AB^	ns
*Macrotiloma axilare*	672 ^B^	673 ^AB^	ns
*Stylosanthes guianensis*	700 ^B^	702 ^AB^	ns
*SEM* ^(1)^	2.44	2.05	2.35
p-values ^(2)^	***	***	
**Partitioning factor (mg DMD/mL)**	**Without PEG**	**With PEG**	**Plant × PEG**
*Cajanus cajan*	3.09 ^§BC^	2.84 ^C^	ns
*Cynodon* spp (Tifton 85)	2.73 ^C^	2.64 ^C^	ns
*Cratylia argentea*	3.82 ^aB^	2.87 ^bC^	**
*Desmodium ovalifolium*	3.11 ^BC^	2.81 ^C^	ns
*Desmodium paniculatum*	5.50 ^aA^	2.14 ^bD^	***
*Flemingia macrophylla*	3.86 ^aB^	2.73 ^bC^	***
*Gliricidia sepium*	3.61 ^BC^	3.39 ^AB^	ns
*Lespedeza procumbens*	4.74 ^aA^	2.28 ^bD^	***
*Leucaena leucocephala*	3.73 ^B^	3.49 ^A^	ns
*Medicago sativa*	3.27 ^BC^	3.32 ^AB^	ns
*Macrotiloma axilare*	3.46 ^BC^	3.36 ^AB^	ns
*Stylosanthes guianensis*	3.22 ^BC^	3.18 ^B^	ns
*SEM* ^(1)^	0.13	0.07	0.24
p-values ^(2)^	***	***	
**NNH_3_ (mg/100 mL)**	**Without PEG**	**With PEG**	**Plant × PEG**
*Cajanus cajan*	22.70 ^§CD^	27.87 ^AB^	ns
*Cynodon* spp (Tifton 85)	22.12 ^CD^	19.37 ^D^	ns
*Cratylia argentea*	25.87 ^BCD^	29.87 ^AB^	ns
*Desmodium ovalifolium*	12.75 ^bF^	20.50 ^aCD^	*
*Desmodium paniculatum*	15.50 ^bF^	30.25 ^aAB^	***
*Flemingia macrophylla*	17.25 ^bEF^	27.87 ^aAB^	***
*Gliricidia sepium*	30.50 ^AB^	34.00 ^A^	ns
*Lespedeza procumbens*	12.37 ^bF^	24.75 ^aBCD^	***
*Leucaena leucocephala*	21.25 ^bDE^	29.12 ^aAB^	*
*Medicago sativa*	32.12 ^A^	26.62 ^BC^	ns
*Macrotiloma axilare*	25.62 ^BCD^	23.37 ^BCD^	ns
*Stylosanthes guianensis*	26.87 ^BC^	27.75 ^AB^	ns
*SEM* ^(1)^	0.82	1.07	2.49
p-values ^(2)^	***	***	

IVDM = in vitro dry matter degradability; IVOMD = in vitro organic matter degradability; DMD = dry matter degradability; PEG = polyethylene glycol; ^(1)^ SEM = standard error of the mean; ^(2)^ ns = not significant (p > 0.05); * p ≤ 0.05; ** p ≤ 0.01; *** p ≤ 0.001. ^§^ Means not followed by the same lowercase letter within rows and means not followed by the same uppercase letter within columns differ (Duncan test at 5%).

**Table 2:** Effects of tannins from plant species on short-chain fatty acids (concentrations at 96 h of incubation in vitro.

Short-Chain Fatty Acid/Plant			
**Acetic Acid (mmol/g OMD)**	**Without PEG**	**With PEG**	**Plant × PEG ^(2)^**
*Cajanus cajan*	8.42 ^§^^AB^	8.43 ^BC^	ns
*Cynodon* spp (Tifton 85)	6.71 ^B^	6.96 ^C^	ns
*Cratylia argentea*	6.74 ^B^	7.55 ^C^	ns
*Desmodium ovalifolium*	8.44 ^AB^	8.03 ^BC^	ns
*Desmodium paniculatum*	8.56 ^bAB^	10.50 ^aA^	**
*Flemingia macrophylla*	9.45 ^A^	9.70 ^AB^	ns
*Gliricidia sepium*	7.20 ^B^	7.52 ^C^	ns
*Lespedeza procumbens*	7.48 ^bAB^	10.48 ^aA^	***
*Leucaena leucocephala*	7.61 ^AB^	6.90 ^C^	ns
*Medicago sativa*	7.52 ^AB^	7.15 ^C^	ns
*Macrotiloma axilare*	6.59 ^B^	6.77 ^C^	ns
*Stylosanthes guianensis*	7.45 ^AB^	7.41 ^C^	ns
*SEM* ^(1)^	0.25	0.17	0.56
p-values ^(2)^	***	***	
**Propionic Acid (mmol/g OMD)**	**Without PEG**	**With PEG**	***Plant* × *PEG***
*Cajanus cajan*	2.35 ^§^^ABC^	2.33 ^BC^	ns
*Cynodon* spp (Tifton 85)	2.29 ^ABC^	2.54 ^AB^	ns
*Cratylia argentea*	2.23 ^ABC^	2.78 ^AB^	ns
*Desmodium ovalifolium*	1.75 ^BC^	2.29 ^BC^	ns
*Desmodium paniculatum*	1.98 ^BC^	2.63 ^AB^	ns
*Flemingia macrophylla*	2.55 ^AB^	2.85 ^A^	ns
*Gliricidia sepium*	2.95 ^A^	3.01 ^A^	ns
*Lespedeza procumbens*	1.56 ^bC^	3.02 ^aA^	***
*Leucaena leucocephala*	2.62 ^AB^	2.30 ^BC^	ns
*Medicago sativa*	1.99 ^BC^	1.93 ^C^	ns
*Macrotiloma axilare*	2.33 ^ABC^	2.31 ^BC^	ns
*Stylosanthes guianensis*	1.96 ^BC^	1.95 ^C^	ns
*SEM* ^(1)^	0.09	0.07	0.24
p-values ^(2)^	*	***	
**Butyric acid (mmol/g OMD)**	**Without PEG**	**With PEG**	**Plant × PEG**
*Cajanus cajan*	0.92 ^§^^ABC^	1.09 ^AB^	ns
*Cynodon* spp (Tifton 85)	1.05 ^A^	1.11 ^AB^	ns
*Cratylia argentea*	0.77 ^DE^	0.89 ^CDE^	ns
*Desmodium ovalifolium*	0.73 ^bDEF^	0.94 ^aBCD^	*
*Desmodium paniculatum*	0.57 ^bFG^	1.14 ^aA^	***
*Flemingia macrophylla*	0.70 ^bDEF^	1.08 ^aAB^	***
*Gliricidia sepium*	0.73 ^DEF^	0.77 ^DE^	ns
*Lespedeza procumbens*	0.45 ^bG^	1.20 ^aA^	***
*Leucaena leucocephala*	0.64 ^EF^	0.74 ^E^	ns
*Medicago sativa*	1.00 ^AB^	0.96 ^BC^	ns
*Macrotiloma axilare*	0.86 ^BCD^	0.87 ^CDE^	ns
*Stylosanthes guianensis*	0.82 ^DC^	0.79 ^CDE^	ns
*SEM* ^(1)^	0.03	0.03	0.06
p-values ^(2)^	***	***	
**Isobutyric acid (mmol/g OMD)**	**Without PEG**	**With PEG**	**Plant × PEG**
*Cajanus cajan*	0.19 ^§^^bABC^	0.26 ^aCDE^	*
*Cynodon* spp (Tifton 85)	0.14 ^BCD^	0.14 ^F^	ns
*Cratylia argentea*	0.21 ^ABC^	0.25 ^CDE^	ns
*Desmodium ovalifolium*	0.17 ^ABCD^	0.19 ^EF^	ns
*Desmodium paniculatum*	n.d ^b^	0.37 ^aA^	***
*Flemingia macrophylla*	0.12 ^bCD^	0.35 ^aAB^	***
*Gliricidia sepium*	0.26 ^A^	0.26 ^CDE^	ns
*Lespedeza procumbens*	0.06 ^bD^	0.33 ^aABC^	***
*Leucaena leucocephala*	0.16 ^bABCD^	0.29 ^aBCD^	*
*Medicago sativa*	0.25 ^AB^	0.24 ^DE^	ns
*Macrotiloma axilare*	0.19 ^ABC^	0.21 ^DEF^	ns
*Stylosanthes guianensis*	0.19 ^ABC^	0.19 ^EF^	ns
*SEM* ^(1)^	0.01	0.01	0.04
p-values ^(2)^	*	***	
**Isovaleric acid (mmol/g OMD)**	**Without PEG**	**With PEG**	**Plant** × **PEG**
*Cajanus cajan*	0.22^§^^bCD^	0.36 ^aBCDE^	***
*Cynodon* spp (Tifton 85)	0.17 ^DE^	0.18 ^H^	ns
*Cratylia argentea*	0.31 ^B^	0.34 ^CDEF^	ns
*Desmodium ovalifolium*	0.14 ^bDEF^	0.24 ^bGH^	***
*Desmodium paniculatum*	0.08 ^bF^	0.52 ^aA^	***
*Flemingia macrophylla*	0.12 ^bEF^	0.44 ^aB^	***
*Gliricidia sepium*	0.29 ^BC^	0.30 ^DEFG^	ns
*Lespedeza procumbens*	0.08 ^bF^	0.41 ^aBC^	***
*Leucaena leucocephala*	0.16 ^bDE^	0.30 ^aDEFG^	***
*Medicago sativa*	0.39 ^A^	0.37 ^BCD^	ns
*Macrotiloma axilare*	0.26 ^BC^	0.28 ^EFG^	ns
*Stylosanthes guianensis*	0.28 ^BC^	0.27 ^FG^	ns
*SEM* ^(1)^	0.02	0.01	0.03
p-values ^(2)^	***	***	
**Valeric acid (mmol/g OMD)**	**Without PEG**	**With PEG**	**Plant × PEG**
*Cajanus cajan*	0.22 ^§^^bAB^	0.28 ^aB^	*
*Cynodon* spp (Tifton 85)	0.18 ^BC^	0.19 ^DE^	ns
*Cratylia argentea*	0.17 ^CD^	0.20 ^DE^	ns
*Desmodium ovalifolium*	0.14 ^bD^	0.19 ^aE^	*
*Desmodium paniculatum*	0.08 ^bE^	0.23 ^aBCD^	***
*Flemingia macrophylla*	0.19 ^bBC^	0.31 ^aA^	***
*Gliricidia sepium*	0.20 ^BC^	0.21 ^CDE^	ns
*Lespedeza procumbens*	0.08 ^bE^	0.23 ^aBCDE^	***
*Leucaena leucocephala*	0.13 ^bD^	0.21 ^aCDE^	***
*Medicago sativa*	0.21 ^ABC^	0.21 ^CDE^	ns
*Macrotiloma axilare*	0.22 ^AB^	0.22 ^BCDE^	ns
*Stylosanthes guianensis*	0.24 ^A^	0.25 ^BC^	ns
*SEM* ^(1)^	0.01	0.01	0.01
p-values ^(2)^	***	***	

^(1)^ SEM = standard error of the mean; ^(2)^ ns = not significant (p > 0.05); * p ≤ 0.05; ** p ≤ 0.01; *** p ≤ 0.001. ^§^ Means not followed by the same lowercase letter within rows and means not followed by the same uppercase letter within columns differ (Duncan test at 5%). PEG = polyethylene glycol; nd = not detected.

**Table 3:** Kinetic parameters of gas production modeled according to France et al. (1993) [10] of legumes forages incubated with a rumen fluid inoculum.

Model Parameters	*A (mL)*		*L (h/min)*		*T/2 (h)*	
	NON	PEG ^(1)^	NON	PEG	NON	PEG
*Cajanus cajan*	122.87 ^§^^bF^	138.50 ^aF^	3.66 ^B^	3.30 ^A^	29.20 ^CD^	23.10 ^D^
*Cynodon* spp	254.52 ^bA^	258.82 ^aA^	4.10 ^AB^	4.00 ^A^	29.56 ^CD^	29.81 ^A^
*Cratylia argentea*	139.45 ^bE^	146.15 ^aF^	3.55 ^B^	4.74 ^A^	27.17 ^DE^	26.30 ^BC^
*Desmodium ovalifolium*	143.12 ^bE^	155.60 ^aE^	4.84 ^AB^	4.18 ^A^	41.45 ^aAB^	27.16 ^bB^
*Desmodium paniculatum*	46.43 ^bH^	145.40 ^aF^	4.47 ^AB^	4.53 ^A^	24.53 ^DEF^	26.37 ^BC^
*Flemingia macrophylla*	81.99 ^bG^	123.27 ^aG^	3.87 ^B^	4.25 ^A^	44.92 ^aA^	29.42 ^bA^
*Gliricidia sepium*	165.92 ^D^	166.42 ^D^	3.92 ^B^	4.47 ^A^	22.25 ^EF^	22.90 ^D^
*Lespedeza procumbens*	75.60 ^bG^	163.85 ^aD^	6.21 ^bA^	3.86 ^aA^	39.42 ^aB^	26.62 ^bBC^
*Leucaena leucocephala*	147.32 ^bE^	165.47 ^aD^	4.72 ^AB^	4.25 ^A^	33.49 ^C^	25.11 ^C^
*Medicago sativa*	188.01 ^aBC^	187.15 ^bC^	3.04 ^B^	3.41 ^A^	20.85 ^F^	21.34 ^E^
*Macrotiloma axilare*	175.25 ^bCD^	180.95 ^aC^	3.53 ^aB^	3.86 ^A^	23.39 ^EF^	23.61 ^D^
*Stylosanthes guianensis*	192.92 ^bB^	197.45 ^aB^	3.38 ^aB^	3.61 ^A^	20.21 ^F^	20.72 ^E^
p-values ^(2)^	***	***	***	***	***	***

^(1)^ PEG = polyethylene glycol; NON = without PEG; ^(2)^ ns = not significant; (p > 0.05); * p ≤ 0.05; ** p ≤ 0.01; *** p ≤ 0.001. ^§^Means not followed by the same lowercase letter within rows and means not followed by the same uppercase letter within columns differ (Duncan test at 5%). France (1993): p = a + b (1 – exp^−ct^), where p = gas production (mL), in time *t*, *a* and *b* = constants of model, *c* = production gas rate (h^−1^), a + b = potential gas production (mL), *L* = lag time, *A* = potential of gas production and *T/2* = asymptotic value.

**Table 4:** Chemical composition of substrates used in the in vitro bioassay (g/kg dry matter (DM)).

Plant	CP	EE	NDF	ADF	ADL	TP ^(1)^	TT ^(1)^	CT ^(2)^
*Cajanus cajan*	200	38	552	438	191	50	39	34
*Cynodon* spp (Tifton 85)	138	13	743	306	133	n.d ^§^.	n.d	n.d
*Cratylia argentea*	195	18	577	418	164	36	21	1.0
*Desmodium ovalifolium*	112	12	614	490	176	237	223	164
*Desmodium paniculatum*	162	20	515	414	216	171	154	70
*Flemingia macrophylla*	189	16	577	465	229	181	168	109
*Gliricidia sepium*	281	23	441	277	130	45	28	0.3
*Lespedeza procumbens*	141	20	475	349	161	108	87	198
*Leucaena leucocephala*	263	22	444	271	136	258	229	48
*Medicago sativa*	271	84	448	245	127	14	8	0.2
*Macrotiloma axilare*	208	22	523	377	105	30	24	1.2
*Stylosanthes guianensis*	212	31	509	350	95	63	41	6.4

DM = dry matter; CP = crude protein; EE = ether extract; NDF = neutral-detergent fiber; ADF = acid-detergent fiber; ADL = acid-detergent lignin; TP = total phenols; TT = total tannins; CT = condensed tannins. ^(1)^ Expressed as equivalent (eq.) g tannic acid/kg DM; ^(2)^ expressed as eq. g leucocyanidin/kg DM; ^§^ n.d.: not detected.
